# Measurement of thermal sweating at rest and steady-state exercise in healthy adults: Inter-day reliability and relationships with components of partitional calorimetry

**DOI:** 10.1371/journal.pone.0278652

**Published:** 2022-12-01

**Authors:** Jennifer S. Peel, Melitta A. McNarry, Shane M. Heffernan, Venturino R. Nevola, Liam P. Kilduff, Mark Waldron

**Affiliations:** 1 Faculty of Science and Engineering, A-STEM Centre, Swansea University, Swansea, United Kingdom; 2 Defence Science and Technology Laboratory (Dstl), Fareham, Hampshire, United Kingdom; 3 Welsh Institute of Performance Science, Swansea University, Swansea, United Kingdom; 4 School of Health and Behavioural Sciences, University of the Sunshine Coast, Queensland, Australia; United States Olympic & Paralympic Committee, UNITED STATES

## Abstract

**Objective:**

Inter-day reliability of sweat measurements, including the absorbent patch and modified iodine-paper techniques, at rest and exercise were evaluated. We further evaluated the effect of iodine paper size and the method of establishing sweat gland activation (sweat gland counting or surface area covered) on reliability. Furthermore, the relationships between all measurement techniques and metabolic heat production [Ḣ_prod_] and evaporative requirement for heat balance [Ė_req_] were determined.

**Method:**

Twelve participants were assessed for whole-body sweat loss (WBSL), local sweat rate (LSR; absorbent patch) and sweat gland activation (SGA; iodine-paper) during rest and sub-maximal cycling at ~200, ~250 and ~300 W/m^2^ Ḣ_prod_ in the heat. Variations in iodine paper (1 x 1 cm-9 x 9 cm) were used to quantify SGA by counting sweat glands or surface area covered. The ‘optimal’ area of SGA was also determined based on the highest density of recruited glands.

**Results:**

All measures of the sweating response were positively related with Ḣ_prod_ and Ė_req_ (*r* = 0.53–0.84), with the 9 x 9 cm and 6 x 6 cm iodine paper sizes being the strongest (*r* = 0.66–0.84) for SGA. Superior inter-day reliability was found for all measures during exercise (CV% = 6–33.2) compared to rest (CV% = 33.5–77.9). The iodine-paper technique was most reliable at 9 x 9 cm (CV% = 15.9) or when the 1 x 1 cm (CV% = 17.6) and 3 x 3 cm (CV% = 15.5) optimal SGA was determined, particularly when measuring the sweat gland number.

**Significance:**

WBSL, LSR and SGA measurement techniques are sufficiently reliable to detect changes in thermal sweating typically reported. We recommend 9 x 9 cm paper sizes or 1 x 1 cm-3 x 3 cm optimal areas, using either gland counting or surface area to determine SGA.

## Introduction

Evaporation of fluid off the skin’s surface represents the greatest avenue of heat loss during exercise in hot environments [1]; eccrine sweat production and evaporation is therefore the most important physiological mechanism for the maintenance of heat balance in such conditions. Indeed, the requirement for evaporative cooling to dissipate excess heat from the body in order to maintain heat balance (Ė_req_), by definition, drives the steady-state sweating response [2–4]. This is supported by a recent study which found that steady-state sweating was similar across a range of absolute core and skin temperature conditions that elicited the same Ė_req_ [5]. Similarly, metabolic heat production (Ḣ_prod_) is also positively related to the rate of whole-body sweating [6], which is intuitive as Ė_req_ is determined as the net difference between Ḣ_prod_ and the sum of respiratory and dry heat exchange [7]. Therefore, both Ḣ_prod_ and Ė_req_ are two modifiable variables responsible for driving the rate of evaporative cooling, which can be estimated using partitional calorimetry in a controlled laboratory setting [7]. Valid and reliable measurements of thermal sweating during exercise, and their determining factors (Ḣ_prod_ and Ė_req_), are fundamental to the investigation and interpretation of human thermoregulation, yet the reliability of these measures has not been sufficiently reported.

There are numerous ways to assess sweating responses in humans [8,9], with many methods designed to determine whole-body or local sweat rate (LSR; [10]) and sweat gland activation (SGA; [11]). Local sweat rate is typically measured using the ventilated capsule method; additionally, absorbent patches affixed to the skin can be used by assessing pre- to post-exercise differences in patch mass, across a known time period [8,10,12]. This is a long-standing technique, which has been adapted from early work in the 1930/40s [13,14], leading to the more recent technical absorbent method [12,15,16] and absorbent patch technique [8,10]. The technical absorbent method was strongly associated with the ventilated capsule method (*r* = 0.74 to 0.95), across varying time durations and patch sizes (sample surface area) and locations (body region; [9]). Estimated whole-body sweat loss (WBSL) is most commonly reported by comparing changes in body mass pre- versus post-exercise [17,18]. Sweat gland activation can be assessed using the modified iodine-paper technique, which is a manual way of measuring sweat droplets on the skin surface and can subsequently be quantified using a computer programme. This technique produces repeatable, intra-trial values (coefficient of variation [CV%] = 11 ± 10%) during controlled exercising conditions [11]. However, this method does not account for clustering of sweat droplets from multiple glands. This could affect the consistency of results and might be resolved by expressing SGA according to the surface area covered, which has not been previously reported in the literature. Lastly, an important element of this technique is the application of iodine impregnated paper onto the skin surface. Varying sizes of iodine paper have been used in different studies and are reported inconsistently [19–23]. It is possible that the size of the paper significantly affects the repeatability of this manual technique, which assumes an even distribution of sweat gland activation across the measured areas. Thus, the effect of the paper size on measurement error requires further investigation.

While intra-trial (i.e. within a single trial day, recorded seconds-to-minutes apart) reliability studies have been conducted for some methods, such as the modified iodine-paper technique [11] and the absorbent patch technique for measuring LSR [10], none have analysed their inter-day reliability at rest and a range of relative exercise intensities. Considering that the majority of studies have multiple testing sessions, spanning several days (or weeks) and exercise intensity varies across studies, it is important to appreciate the variability under such differing circumstances and the subsequent reliability of the technique. In addition, there are daily fluctuations in individuals’ physiological responses to stimuli, which could potentially create noise when assessments are conducted across a longer time period [24]. Without quantifying the reliability (i.e. noise) of a technique across several days, its efficacy (e.g. detection limits) is unknown, and thus the capacity to identify meaningful changes in sweat (i.e. rate and SGA) as a result of an intervention, such as acclimation or dietary supplementation, cannot be determined [25]. Furthermore, the reliability of these techniques should be considered in conjunction with the established drivers of thermal sweating (Ḣ_prod_ and Ė_req_) since error in sweating measures will be partly determined by variation in these factors.

On the understanding that Ḣ_prod_ and Ė_req_ predominantly determine the magnitude of required evaporative cooling during exercise in hot environments [4,5], it stands to reason that any measurement used to determine the sweating response should be positively associated, and therefore constructively valid. This includes measures such as SGA, LSR and WBSL. Indeed, Ḣ_prod_ and Ė_req_ have been positively associated to whole-body sweat rate and LSR, determined using absorbent patches [26]. The relationship between Ė_req_ and whole-body sweat rate, measured via the ventilated capsule method has also been established [4–6]. However, the relationship between Ḣ_prod_ and Ė_req_ and all other techniques for assessing the sweating response have not.

Based on the above reasoning, the aims of the current study were to establish the inter-day reliability of: i) the modified iodine-paper technique for the measurement of SGA using two separate assessment methods (sweat gland counting and surface area covered); ii) the absorbent patch technique for the measurement of LSR; and iii) pre- vs post-exercise body mass changes for measurement of WBSL. Finally, the relationship between all measurements of thermal sweating and both Ḣ_prod_ and Ė_req_ was assessed to establish the construct validity of these measurements.

## Methods

### Participants

Twelve, non-heat acclimated, healthy, recreationally active, females (*n* = 5) and males (*n* = 7) volunteered for the study (29 ± 6 years, 175.0 ± 7.6 cm, 76.5 ± 11.6 kg). Participants were asked to refrain from alcohol, avoid strenuous exercise and follow a consistent diet for 24-h prior to testing. Use of any dietary supplement was prohibited for the duration of the study. Written informed consent was obtained from all participants. Institutional ethical approval was provided for this study, which was conducted in accordance with the 2013 Declaration of Helsinki.

### Design

Participants reported to the laboratory on three occasions, across three separate days. The first visit comprised of preliminary testing and familiarisation; the inter-day test-retest reliability trials were subsequently conducted on the second and third visits. Specifically, during visits 2 and 3, the participants completed a discontinuous, sub-maximal cycling protocol at exercise intensities designed to elicit three 30-min stages of incremental heat production (Ḣ_prod_; ~200, ~250 and ~300 W/m^2^) whilst exposed to ambient heat stress. Each stage was separated by 10-min of rest. During each stage sweat-related measurements were conducted for subsequent assessment of their reliability between days. All visits took place in a temperature-controlled room at 37.6 ± 0.4°C and 27.0 ± 5.9% RH (relative humidity; mean ± SD). To minimise acclimation effects and permit recovery between visits, all exercise trials were separated by four days [27]. For women, all tests were conducted in the same phase of the menstrual cycle, determined by self-reporting using a day counting method [28]. All trials were conducted at the same time of day to control for circadian variation. To limit between-investigator error whilst performing measurement techniques, the same member of the research team conducted all trials.

### Preliminary testing

During visit 1, participants undertook an incremental exercise test to volitional exhaustion on a cycle ergometer (Monark Exercise AB, Ergomedic 874E, Varberg, Sweden) in hot ambient conditions (37.6 ± 0.4°C and 27.0 ± 5.9% RH) to determine their individual work rates required to elicit Ḣ_prod_ of ~200, ~250 and ~300 W/m^2^ and their peak oxygen consumption ($\dot{V}O_{2\mathrm{peak}}$). Participants performed a 5-min warm-up at 80 W, followed by 5-min rest, before commencement of the exercise test. Oxygen consumption ($\dot{V}O_{2}$) was measured using breath-by-breath gas analysis (Jaeger Vyntus CPX, Hoechberg, Germany), with $\dot{V}O_{2\mathrm{peak}}$ determined as the highest 30-s mean value, which occurred in the final stage of each participant’s test. Criteria for achieving $\dot{V}O_{2\mathrm{peak}}$ was: 1) reaching volitional exhaustion; 2) respiratory exchange ratio (RER) > 1.15; 3) final HR within 10 beats/min of age-predicted maximum; and 4) rating of perceiving exertion (RPE) > 19 (6–20 Borg scale; [29]). The test was designed to progressively increase mechanical work rate on the ergometer, in a square-wave manner, to elicit a range of Ḣ_prod_ values, including those required for exercise in visits 2 and 3. The Ḣ_prod_ was determined by subtracting the rate of mechanical work (Wk) from the rate of metabolic energy expenditure (Ṁ; Eq 1).


$${Ḣ}_{prod}=Ṁ-Wk\;[W]$$ (Eq 1)

Where metabolic energy expenditure (Ṁ) was determined using measured $\dot{V}O_{2}$ and $\dot{V}CO_{2}$ within the final 1-min of each stage (Eq 2):

$$Ṁ={\dot{V}O}_{2}\times \frac{\left((\frac{RER-0.7}{0.3})\times 21.13)+((\frac{1.0-RER}{0.3})\times 19.62\right)}{60}\times 1000\;[W]$$ (Eq 2)

To achieve the necessary Ḣ_prod_ values, the test was initiated at a mechanical work rate below that which was required to elicit the lowest desired Ḣ_prod_ (200 W/m^2^) and increased by 20 W every 5-min at a fixed cadence of 80 rpm until exhaustion. The Ḣ_prod_ (W/m^2^) at each stage was estimated based on participant body surface area (BSA; Eqs 3 and 4; [2]).


$${Ḣ}_{prod}=\frac{{Ḣ}_{prod}}{BSA}\;[W/m^{2}]$$ (Eq 3)

$$BSA=0.00718\times ({body\;mass\;(kg)}^{0.425})\times ({height\;(cm)}^{0.725})[m^{2}];$$ (Eq 4)

Dubois & Dubois equation [30].

The mechanical work rate required to elicit each target Ḣ_prod_ (W/m^2^) for the exercise trials during visits 2 and 3 (i.e. ~200, ~250 and ~300 W/m^2^) was subsequently estimated based on the linear relationship (y = a + b), between Ḣ_prod_ (W/m^2^) and work rate during the incremental test (Eq 5).


$$Required\;work\;rate=\frac{Desired\;{Ḣ}_{prod}\;(W/m^{2})-y\;intercept}{Slope}\;[W]$$ (Eq 5)

### Inter-day test-retest reliability

#### Pre-exercise instrumentation

Participants were required to arrive euhydrated, as determined by a urine osmolality value < 600 mOsm kg/H_2_O (Portable osmometer, Osmocheck, Vitech, Scientific Ltd). If the participant was not deemed to be euhydrated, they were asked to drink 500-ml of plain water and wait 30-min before their urine osmolality was re-measured. Participants wore standardised cycling shorts (94% polyester; 6% elastane), as well as a sports bra for female participants. To measure core body temperature (T_core_), participants were instructed to insert a flexible rectal probe 10 cm past the anal sphincter. Skin thermistors (Grant Instruments Ltd., Cambridge, UK) were attached to four sites on the participant’s right side: upper-chest, mid-humerus, mid-calf and mid-thigh to measure mean skin temperature. Ramanathan’s equation [31] was used to calculate mean skin temperature:

$$T_{sk}=(T_{chest}+T_{arm})\times 0.3+(T_{thigh}+T_{calf})\times 0.2\;[{}^{\circ}C]$$ (Eq 6)

Prior to application of the skin thermistors, the skin was dry-shaved, cleaned with soap and water and allowed to air-dry. Both core and skin temperature were continuously recorded using a data logger (SQ2010; Grant Instruments Ltd., Cambridge, UK). Heart rate (HR) was continuously recorded throughout each exercise trial (Polar Heart Rate Monitor M400, Warwick, UK). Each participant’s body mass was measured (whilst wearing cycling shorts, a sports bra for women, the HR monitor, the inserted rectal thermistor and the skin thermistors) using a calibrated scale (resolution 50 g; Seca 711, Hamburg, Germany). This was necessary because of the repeated body mass measurements throughout the trial.

#### Exercise trials

Participants initially rested for 30-mins in a seated position in a temperature-controlled room that was regulated to an air temperature of 37.6 ± 0.4°C and a %RH of 27.0 ± 5.9%. Environmental conditions, such as ambient dry bulb temperature (°C), RH and air velocity (m/s), were continuously monitored (Kestrel 5400 Heat Stress Tracker, Kestrel Meters, Boothwyn, PA, US). An electric fan (SIP 24” Drum Fan, Loughborough, UK) was placed adjacent to the participant during the rest period and diagonally in-front during the exercise periods, providing an airflow of 1 m/s directed at the torso. After 30-min of rest, the participants were seated on the ergometer and performed three exercising periods of 30-min, at the three pre-determined fixed rates of Ḣ_prod_ (~200, ~250 & ~300 W/m^2^). During exercise, oxygen consumption was measured using the same breath-by-breath expired gas analyser (Jaeger Vyntus CPX, Hoechberg, Germany). Participants’ body mass was measured, and they were provided with 200-ml of plain water (maintained at room temperature [~20°C]) to drink between each 30-min period. Rating of perceived exertion was recorded using a 6–20 point Borg scale [29], while thermal comfort (TC) was recorded using a 7-point scale (where -3 = “much too cool”, 0 = “comfortable” and 3 = “much too warm” [32]). Thermal sensation (TS) was recorded using a 9-point scale (where -4 = “very cold”, 0 = “neutral” and 4 = “very hot” [33]). All perceptual data (RPE, TC and TS) were recorded at 5-min intervals during the rest and exercise periods, and upon completion of the trial. Local sweat rate and SGA measurements were taken using the techniques described below.

#### Partitional calorimetry

As detailed in supplementary material, heat balance parameters such as Ḣ_prod_ and Ė_req_ (Eq 7) were estimated for each 30-min exercise period via partitional calorimetry [7]. Ḣ_prod_ was also expressed relative to body surface area [30].


$${Ė}_{req}={Ḣ}_{prod}-{Ḣ}_{dryskin}-{Ḣ}_{res}\;[W]$$ (Eq 7)

#### Local sweat rate measurement

Local sweat rate was determined using the absorbent patch technique [10], on the left scapula. Measurements were taken during the final 5-min of each 30-min exercising period. Prior to the trial, the area of interrogation on the skin’s surface was shaved and cleaned using water and gauze. A template matching the size of the absorbent patch was pressed to the skin’s surface and outlined in indelible ink to identify the area of measurement and ensure consistency of application across the exercise stages and the two inter-day reliability trials. The patch (Medipore + Pad [3M]) was 5 cm x 5.5 cm, with an absorbent capacity of ~7 g. Immediately before patch application, the skin was wiped dry with gauze. The patch was weighed (resolution 0.01 g; Ohaus, Navigator N24120, Nänikon Switzerland) prior to and after the 5-min skin application. Local sweat rate (mg/cm^2^/min) was determined as: pre vs post change in patch mass in milligrams, divided by the surface area of the patch (5 cm x 5.5 cm) and the duration of application (5-min; Eq 8).


$$Local\;sweat\;rate=\frac{pre\;to\;post\;change\;in\;patch\;mass\;(mg)}{\left[5\;(cm)\times 5.5\;(cm)]\times 5\;(min\right)}[mg/{cm}^{2}/min]$$ (Eq 8)

#### Modified iodine-paper technique

**Fig 1:**
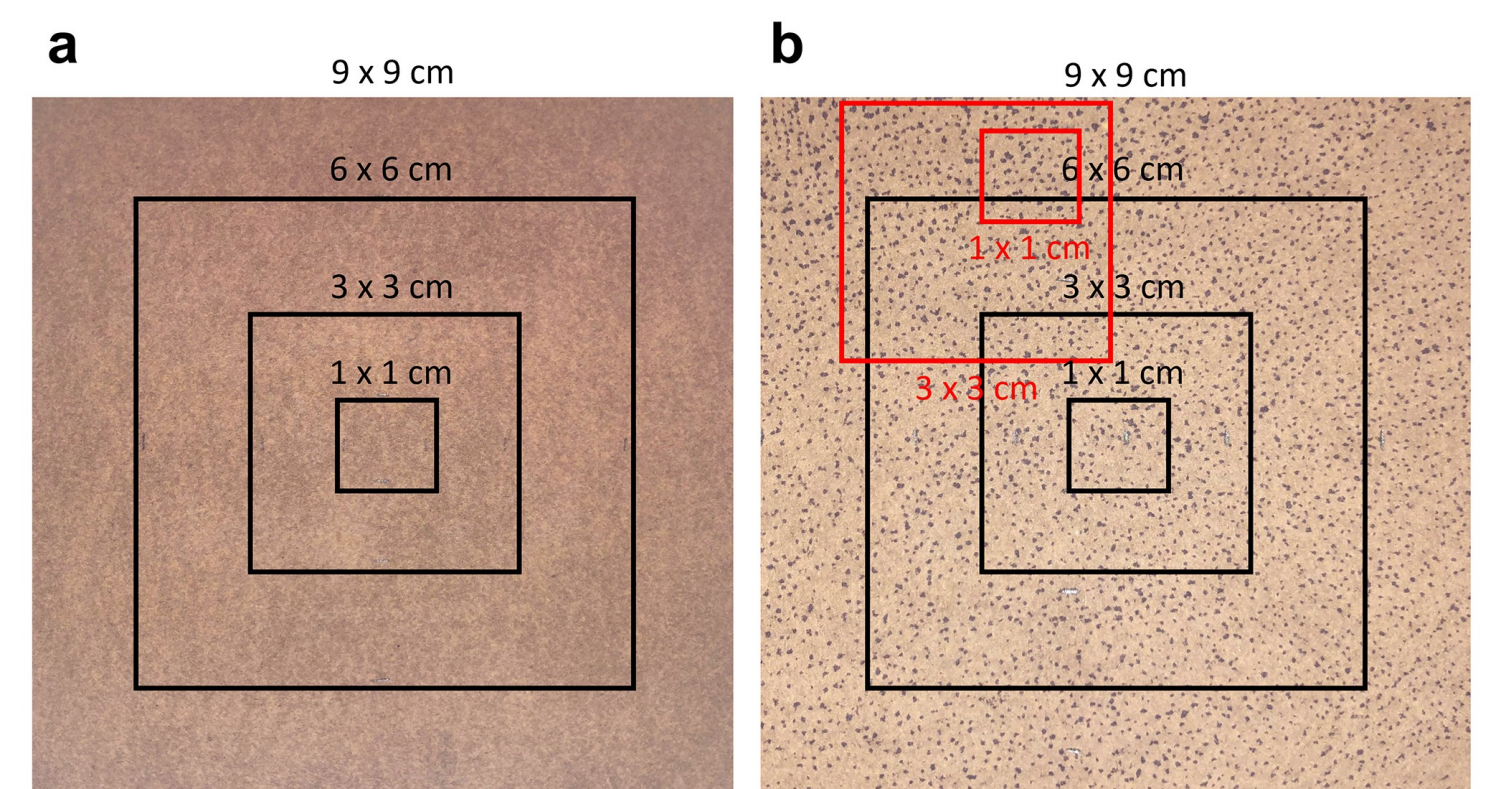
(**a**) Cotton paper divided into pre-determined areas and (**b**) Iodine impregnated paper displaying sweat gland activation within the pre-determined and optimal (red) areas.

The modified iodine-paper technique [11] was used to determine SGA on the right scapula. 100% cotton paper (Southworth, Agawam, MA, US) was cut to 9 x 9 cm and further divided into 6 x 6 cm, 3 x 3 cm and 1 x 1 cm sections using a fine-point pencil (Fig 1). The paper was placed in an air-tight sealed box, containing iodine in solid form (Sigma-Aldrich, St. Louis, MO). Each 9 x 9 cm piece of cotton paper was suspended from the lid of the container to avoid direct contact with the iodine. The pieces of paper were impregnated with iodine after ~48-h, after which they were removed and placed in sealed bags. Double-sided tape was used to affix the cotton paper to a hard-flat plastic surface to ensure uniform application to the skin. Prior to testing, a 9 x 9 cm template was pressed to the skin’s surface at the designated site and outlined in indelible ink to ensure consistency of application across the exercise stages and the inter-day trials. At the end of the rest period and each 30-min exercise period, the skin’s surface was blotted dry using a small towel before the iodine-impregnated cotton paper was firmly applied for 5-s. Visually identifiable blue colourations formed on the paper, indicating excreted sweat from active sweat glands during the 5-s application (Fig 1). A high-resolution (3024 x 4032) photograph (image) was taken of the paper and subsequently analysed using ImageJ [34].

#### Iodine paper image processing and analysis

ImageJ was used to determine i) the number of active sweat glands and ii) the percentage surface area covered with sweat within each pre-determined section of the paper (9 x 9 cm, 6 x 6 cm, 3 x 3 cm, 1 x 1 cm). Further analysis was also performed to determine the optimal area of sweat gland density within 3 x 3 cm and 1 x 1 cm areas within the 9 x 9 cm iodine paper area. This was defined as the area (3 x 3 cm and 1 x 1 cm) with the highest density of recruited glands. All images were taken in a well-lit area to avoid colour contrasts on the paper. For the purposes of replication, please see supplementary files for a detailed guide on ImageJ functions. The number of active sweat glands and the percentage surface area covered in each pre-determined area were later normalised to the maximum value for each individual across all exercise periods. This was to account for inter-individual variation in both the maximal SGA and the point of uncompensability (i.e. individual differences in the balance between evaporative heat transfer [Ė_req_] and the maximum evaporative capacity of the environment [Ė_max_]; [7]).

### Statistical analyses

Data were analysed using SPSS (IBM SPSS Statistics for Windows, IBM Corp, Version 24.0. Armonk, New York) and R 4.0.2 (R Core Team, 2018). A 2 (trial 1 and trial 2) x 4 (rest and exercising Ḣ_prod_ levels) factorial analysis of variance (Two-way repeated measures ANOVA) was conducted to evaluate systematic biases (i.e. differences) in Ḣ_prod_, Ė_req_, WBSLs, LSR and SGA (gland number, surface area covered, normalised gland number and normalised surface area covered). Identification of interaction effects and Bonferroni-adjusted tests were planned for analysis of any pair-wise systematic biases [35] in sweat-related measures between trials 1 and 2 at each of the three levels of exercising Ḣ_prod_. The inter-day reliability was further assessed using the coefficient of variation (CV% ± 95% confidence limits; [35]) on each pairwise comparison. To calculate the CV, the SD of the data was divided by the mean and multiplied by 100 [35]. Repeated measures correlations using the *rmcorr* package in R (v0.4.4; Bakdash & Marusich) were used to establish the relationships between Ė_req_ and Ḣ_prod_ with WBSL, LSR and SGA (normalised gland number and surface area covered) at each exercise intensity. The repeated measures factor was the participant, as data from each exercise period were pooled, leading to multiple (three) entries from each participant. Confidence intervals (CI) were bootstrapped and the CI level was set at 95%. The alpha level (ɑ) for the repeated measures correlations was Bonferroni-adjusted to account for the number of correlations under each hypothesis (*n* = 14). Data are expressed as means ± SD throughout and a significance level of *P* < 0.05 was accepted across all tests. The thresholds for the magnitudes of effects for correlations were < 0.2, 0.2, 0.5 and 0.8 for trivial, small, moderate and large effects, respectively [36].

## Results

### Whole-body sweat loss and local sweat rate

Participants’ mean values for WBSL and LSR during both trials and between-trial reliability (CV%) are presented in Table 1. There were no trial main effects or trial x period interaction effects for WBSL (*P* = .816; *P* = .272, respectively) and LSR (*P* = .468; *P* = .439, respectively).

**Table 1:** Mean, standard deviation and reliability of whole-body sweat loss and local sweat rate at rest and a range of exercise intensities.

Variable	Trial 1	Trial 2	CV% ± 95% CI
**Whole-body sweat loss (g)**			
Rest	112 ± 64	108 ± 51	33.5 ± 23.8
200 (W/m^**2**^) Ḣ_**prod**_	363 ± 71	333 ± 75	11.0 ± 3.7
250 (W/m^**2**^) Ḣ_**prod**_	421 ± 81	437 ± 103	6.4 ± 2.8
300 (W/m^**2**^) Ḣ_**prod**_	514 ± 87	523 ± 75	6.0 ± 2.0
All exercise	430 ± 99	429 ± 114	7.8 ± 1.8
Overall	349 ± 167	347 ± 174	14.4 ± 6.8
Local sweat rate (mg/cm^2^/min)			
Rest	0.09 ± 0.15	0.07 ± 0.10	77.9 ± 39.1
200 (W/m^**2**^) Ḣ_**prod**_	1.06 ± 0.53	0.99 ± 0.37	12.8 ± 4.8
250 (W/m^**2**^) Ḣ_**prod**_	1.56 ± 0.60	1.66 ± 0.79	10.1 ± 5.6
300 (W/m^**2**^) Ḣ_**prod**_	1.98 ± 0.89	2.16 ± 1.13	18.8 ± 6.1
All exercise	1.52 ± 0.76	1.59 ± 0.93	13.8 ± 3.3
Overall	1.20 ± 0.90	1.20 ± 1.0	30.1 ± 12.8

Note: “All exercise” is the mean of all work rates combined; Trial 1 & 2 data presented as mean ± standard deviation

CV%, coefficient of variation; CI, confidence interval; Ḣ_prod_, heat production

### Sweat gland activation

Participants’ mean values for SGA (gland number and surface area covered) during both trials and between trial reliability in the form of coefficient of variation (CV%) are presented in Tables 2 & 3. There were no trial main effects or trial x period interaction effects for gland number at 9 x 9 cm (*P* = .085; *P* = .488, respectively), 3 x 3 cm (*P* = .210; *P* = .407, respectively), optimal 3 x 3 cm (*P* = .056; *P* = .526, respectively), 1 x 1 cm (*P* = .125; *P* = .217, respectively) and optimal 1 x 1 cm (*P* = .212; *P* = .115, respectively) and surface area covered at 9 x 9 cm (*P* = .785; *P* = .372, respectively), 6 x 6 cm (*P* = .227; *P* = .357, respectively), 3 x 3 cm (*P* = .709; *P* = .407, respectively), optimal 3 x 3 cm (*P* = .133; *P* = .754, respectively), 1 x 1 cm (*P* = .808; *P* = .282, respectively) and optimal 1 x 1 cm (*P* = .571; *P* = .100, respectively). A significant trial main effect was found for gland number at 6 x 6 cm (*P* = .021), but no trial x period interaction effect (*P* = .571).

**Table 2:** Mean, standard deviation and reliability of sweat gland activation (gland number) at rest and a range of exercise intensities.

Variable	Trial 1	Trial 2	CV% ± 95% CI
**9 x 9 cm gland number**			
Rest	425 ± 435	1,174 ± 1,162	56.1 ± 26.1
200 (W/m^**2**^) Ḣ_**prod**_	3,403 ± 1,707	3,864 ± 1,395	20.1 ± 10.8
250 (W/m^**2**^) Ḣ_**prod**_	4,888 ± 1,500	5,063 ± 1,833	12.8 ± 4.4
300 (W/m^**2**^) Ḣ_**prod**_	6,918 ± 3,228	6,922 ± 2,731	14.7 ± 6.3
All exercise	5,017 ± 2,614	5,236 ± 2,346	15.9 ± 4.5
Overall	3,845 ± 3,032	4,199 ± 2,756	26.2 ± 8.8
**6 x 6 cm gland number**			
Rest	215 ± 199	691 ± 699	61.9 ± 29.8
200 (W/m^**2**^) Ḣ_**prod**_	1,769 ± 906	2,160 ± 817	24.1 ± 11.1
250 (W/m^**2**^) Ḣ_**prod**_	2,468 ± 932	2,592 ± 1,001	14.4 ± 7.5
300 (W/m^**2**^) Ḣ_**prod**_	3,394 ± 1,988	3,588 ± 1,713	15.2 ± 5.5
All exercise	2,519 ± 1,468	2,757 ± 1,327	18.0 ± 5.0
Overall	1,931 ± 1,623	2,229 ± 1,499	29.2 ± 9.9
**3 x 3 cm gland number**			
Rest	62 ± 57	200 ± 213	54.1 ± 32
200 (W/m^**2**^) Ḣ_**prod**_	502 ± 252	627 ± 264	22.9 ± 11.0
250 (W/m^**2**^) Ḣ_**prod**_	690 ± 284	730 ± 290	14.6 ± 7.9
300 (W/m^**2**^) Ḣ_**prod**_	913 ± 639	866 ± 361	19.0 ± 11.4
All exercise	695 ± 442	737 ± 313	18.8 ± 5.8
Overall	534 ± 472	600 ± 373	27.8 ± 10
**Optimal 3 x 3 cm gland number**			
Rest	95 ± 84	237 ± 234	53.7 ± 26.9
200 (W/m^**2**^) Ḣ_**prod**_	617 ± 206	629 ± 292	15.9 ± 10.8
250 (W/m^**2**^) Ḣ_**prod**_	827 ± 236	899 ± 351	11.5 ± 6.6
300 (W/m^**2**^) Ḣ_**prod**_	1,051 ± 666	1,117 ± 657	19.3 ± 10.5
All exercise	825 ± 440	875 ± 485	15.5 ± 5.4
Overall	639 ± 499	712 ± 516	25.2 ± 9.1
**1 x 1 cm gland number**			
Rest	6 ± 6	22 ± 22	70.0 ± 30.5
200 (W/m^**2**^) Ḣ_**prod**_	52 ± 25	68 ± 30	23.3 ± 6.4
250 (W/m^**2**^) Ḣ_**prod**_	75 ± 38	79 ± 35	21.1 ± 12.8
300 (W/m^**2**^) Ḣ_**prod**_	88 ± 41	90 ± 36	22.2 ± 9.3
All exercise	71 ± 37	78 ± 34	22.2 ± 5.5
Overall	55 ± 43	64 ± 40	34.4 ± 10.5
**Optimal 1 x 1 cm gland number**			
Rest	14 ± 12	32 ± 31	51.7 ± 24.0
200 (W/m^**2**^) Ḣ_**prod**_	75 ± 19	87 ± 34	24.1 ± 9.6
250 (W/m^**2**^) Ḣ_**prod**_	110 ± 38	104 ± 32	15.0 ± 7.5
300 (W/m^**2**^) Ḣ_**prod**_	116 ± 42	124 ± 34	13.4 ± 8.4
All exercise	100 ± 38	104 ± 35	17.6 ± 5.0
Overall	78 ± 50	86 ± 47	26.3 ± 8.2

Note: “All exercise” is the mean of all work rates combined; Trial 1 & 2 data presented as mean ± standard deviation.

CV%, coefficient of variation; CI, confidence interval; Ḣ_prod_, heat production.

**Table 3:** Mean, standard deviation and reliability of sweat gland activation (area covered) at rest and a range of exercise intensities.

Variable	Trial 1	Trial 2	CV% ± 95% CI
**9 x 9 cm area covered**			
Rest	0.47 ± 0.56%	0.87 ± 0.90%	41.2 ± 22.8
200 (W/m^**2**^) Ḣ_**prod**_	2.30 ± 1.85%	2.54 ± 1.44%	26.3 ± 11.1
250 (W/m^**2**^) Ḣ_**prod**_	3.92 ± 2.78%	3.89 ± 2.51%	13.8 ± 4.4
300 (W/m^**2**^) Ḣ_**prod**_	6.11 ± 3.68%	5.80 ± 2.95%	20.3 ± 7.6
All exercise	4.05 ± 3.17%	4.03 ± 2.66%	20.1 ± 4.9
Overall	3.14 ± 3.16%	3.22 ± 2.71%	25.5 ± 7.2
**6 x 6 cm area covered**			
Rest	0.44 ± 0.46%	1.00 ± 1.07%	55.7 ± 27.3
200 (W/m^**2**^) Ḣ_**prod**_	2.43 ± 1.92%	2.95 ± 1.85%	33.2 ± 11.5
250 (W/m^**2**^) Ḣ_**prod**_	3.98 ± 2.78%	3.93 ± 2.63%	14.0 ± 7.1
300 (W/m^**2**^) Ḣ_**prod**_	5.95 ± 4.09%	5.81 ± 3.21%	12.6 ± 3.9
All exercise	4.07 ± 3.27%	4.19 ± 2.79%	20.2 ± 5.6
Overall	3.14 ± 3.24%	3.37 ± 2.83%	29.2 ± 9.1
**3 x 3 cm area covered**			
Rest	0.54 ± 0.55%	1.20 ± 1.28%	52.4 ± 28.1
200 (W/m^**2**^) Ḣ_**prod**_	2.71 ± 2.16%	3.43 ± 2.14%	35.4 ± 14.0
250 (W/m^**2**^) Ḣ_**prod**_	4.36 ± 3.11%	4.29 ± 2.81%	16.3 ± 10.1
300 (W/m^**2**^) Ḣ_**prod**_	6.36 ± 4.86%	5.50 ± 3.02%	23.4 ± 12.5
All exercise	4.42 ± 3.72%	4.38 ± 2.73%	25.4 ± 7.5
Overall	3.43 ± 3.64%	3.56 ± 2.81%	32.3 ± 9.5
**Optimal 3 x 3 cm area covered**			
Rest	0.86 ± 0.85%	1.51 ± 1.57%	42.6 ± 26.0
200 (W/m^**2**^) Ḣ_**prod**_	4.16 ± 3.07%	4.55 ± 2.47%	20.5 ± 13.6
250 (W/m^**2**^) Ḣ_**prod**_	5.31 ± 2.39%	5.38 ± 2.58%	16.8 ± 7.1
300 (W/m^**2**^) Ḣ_**prod**_	7.90 ± 4.88%	7.82 ± 4.42%	10.5 ± 4.5
All exercise	5.73 ± 3.79%	5.86 ± 3.43%	16.1 ± 5.5
Overall	4.49 ± 3.92%	4.75 ± 3.60%	22.9 ± 8.3
**1 x 1 cm area covered**			
Rest	0.51 ± 0.42%	1.27 ± 1.34%	62.2 ± 27.2
200 (W/m^**2**^) Ḣ_**prod**_	2.77 ± 1.91%	3.65 ± 2.30%	31.9 ± 11.5
250 (W/m^**2**^) Ḣ_**prod**_	4.93 ± 3.65%	4.81 ± 2.76%	27.4 ± 12.2
300 (W/m^**2**^) Ḣ_**prod**_	6.89 ± 5.75%	5.74 ± 3.14%	30.8 ± 15.8
All exercise	4.80 ± 4.25%	4.70 ± 2.79%	30.0 ± 7.4
Overall	3.71 ± 4.12%	3.83 ± 2.92%	38.2 ± 9.6
**Optimal 1 x 1 cm area covered**			
Rest	0.74 ± 0.67%	1.74 ± 1.73%	48.6 ± 27.1
200 (W/m^**2**^) Ḣ_**prod**_	4.28 ± 2.15%	5.06 ± 2.29%	22.3 ± 12.4
250 (W/m^**2**^) Ḣ_**prod**_	6.50 ± 2.67%	6.13 ± 2.62%	20.2 ± 8.6
300 (W/m^**2**^) Ḣ_**prod**_	9.41 ± 5.72%	8.89 ± 4.81%	12.5 ± 5.9
All exercise	6.66 ± 4.23%	6.63 ± 3.65%	18.5 ± 5.5
Overall	5.15 ± 4.49%	5.38 ± 3.90%	26.2 ± 8.7

Note: “All exercise” is the mean of all work rates combined; Trial 1 & 2 data presented as mean ± standard deviation.

CV%, coefficient of variation; CI, confidence interval; Ḣ_prod_, heat production.

There were also no trial main effects or trial x period interaction effects for normalised gland number at 9 x 9 cm (*P* = .209; *P* = .424, respectively), 3 x 3 cm (*P* = .138; *P* = .459, respectively), optimal 3 x 3 cm (*P* = .442; *P* = .625, respectively), 1 x 1 cm (*P* = .178; *P* = .152, respectively) and optimal 1 x 1 cm (*P* = .484; *P* = .367, respectively) and normalised surface area covered at 9 x 9 cm (*P* = .551; *P* = .456, respectively), 6 x 6 cm (*P* = .123; *P* = .123, respectively), 3 x 3 cm (*P* = .351; *P* = .270, respectively), 1 x 1 cm (*P* = .370; *P* = .325, respectively) and optimal 1 x 1 cm (*P* = .136; *P* = .154, respectively). There was a significant trial main effect, but no trial x period interaction effect for normalised gland number at 6 x 6 cm (*P* = .048; *P* = .362, respectively) and surface area covered at optimal 3 x 3 cm (*P* = .046; *P* = .430, respectively).

### Partitional calorimetry

Participants’ mean values for absolute Ḣ_prod_ (W), Ḣ_prod_ (W/m^2^), Ė_req_ (W) and Ė_req_ (W/m^2^) during both trials and between trial reliability in the form of coefficient of variation (CV%) are presented in Table 4. There were no trial main effects or trial x period interaction effects for Ḣ_prod_ (*P* = .590; *P* = .112, respectively), Ḣ_prod_ (W/m^2^; *P* = .603; *P* = .126, respectively), Ė_req_ (*P* = .904; *P* = .187, respectively) and Ė_req_ (W/m^2^; *P* = .946; *P* = .211, respectively).

**Table 4:** Mean, standard deviation and reliability of partitional calorimetry variables (Ḣ_prod_ and Ė_req_) at a range of exercise intensities.

Variable	Trial 1	Trial 2	CV% ± 95% CI
**Ḣ_prod_ (W)**			
200 (W/m^**2**^) Ḣ_**prod**_	377 ± 48	377 ± 48	2.4 ± 1.1
250 (W/m^**2**^) Ḣ_**prod**_	474 ± 58	472 ± 52	1.5 ± 0.8
300 (W/m^**2**^) Ḣ_**prod**_	564 ± 68	556 ± 64	1.6 ± 0.7
Overall	469 ± 96	466 ± 90	1.8 ± 0.5
**Ḣ_prod_ (W/m^2^)**			
200 (W/m^**2**^) Ḣ_**prod**_	196 ± 19	196 ± 14	2.5 ± 1.1
250 (W/m^**2**^) Ḣ_**prod**_	246 ± 20	246 ± 17	1.5 ± 0.8
300 (W/m^**2**^) Ḣ_**prod**_	295 ± 22	291 ± 19	1.7 ± 0.7
Overall	244 ± 45	243 ± 42	1.9 ± 0.5
**Ė_req_ (W)**			
200 (W/m^**2**^) Ḣ_**prod**_	373 ± 45	375 ± 41	2.2 ± 0.9
250 (W/m^**2**^) Ḣ_**prod**_	463 ± 54	464 ± 50	1.5 ± 0.7
300 (W/m^**2**^) Ḣ_**prod**_	544 ± 66	538 ± 62	1.3 ± 0.8
Overall	457 ± 89	457 ± 84	1.7 ± 0.5
**Ė_req_ (W/m^2^)**			
200 (W/m^**2**^) Ḣ_**prod**_	194 ± 18	195 ± 13	2.2 ± 0.9
250 (W/m^**2**^) Ḣ_**prod**_	241 ± 19	241 ± 17	1.5 ± 0.7
300 (W/m^**2**^) Ḣ_**prod**_	285 ± 22	282 ± 19	1.4 ± 0.8
Overall	239 ± 42	238 ± 39	1.7 ± 0.5

Note: Trial 1 & 2 data presented as mean ± standard deviation.

CV%, coefficient of variation; CI, confidence interval; Ḣ_prod_, heat production; Ė_req_, evaporative requirement for heat balance.

### Correlations

**Fig 2:**
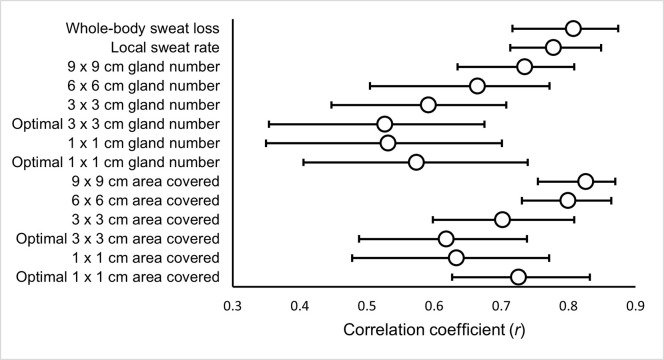
The relationship between absolute heat production (Ḣ_prod_) and measures of the sweating response.

**Fig 3:**
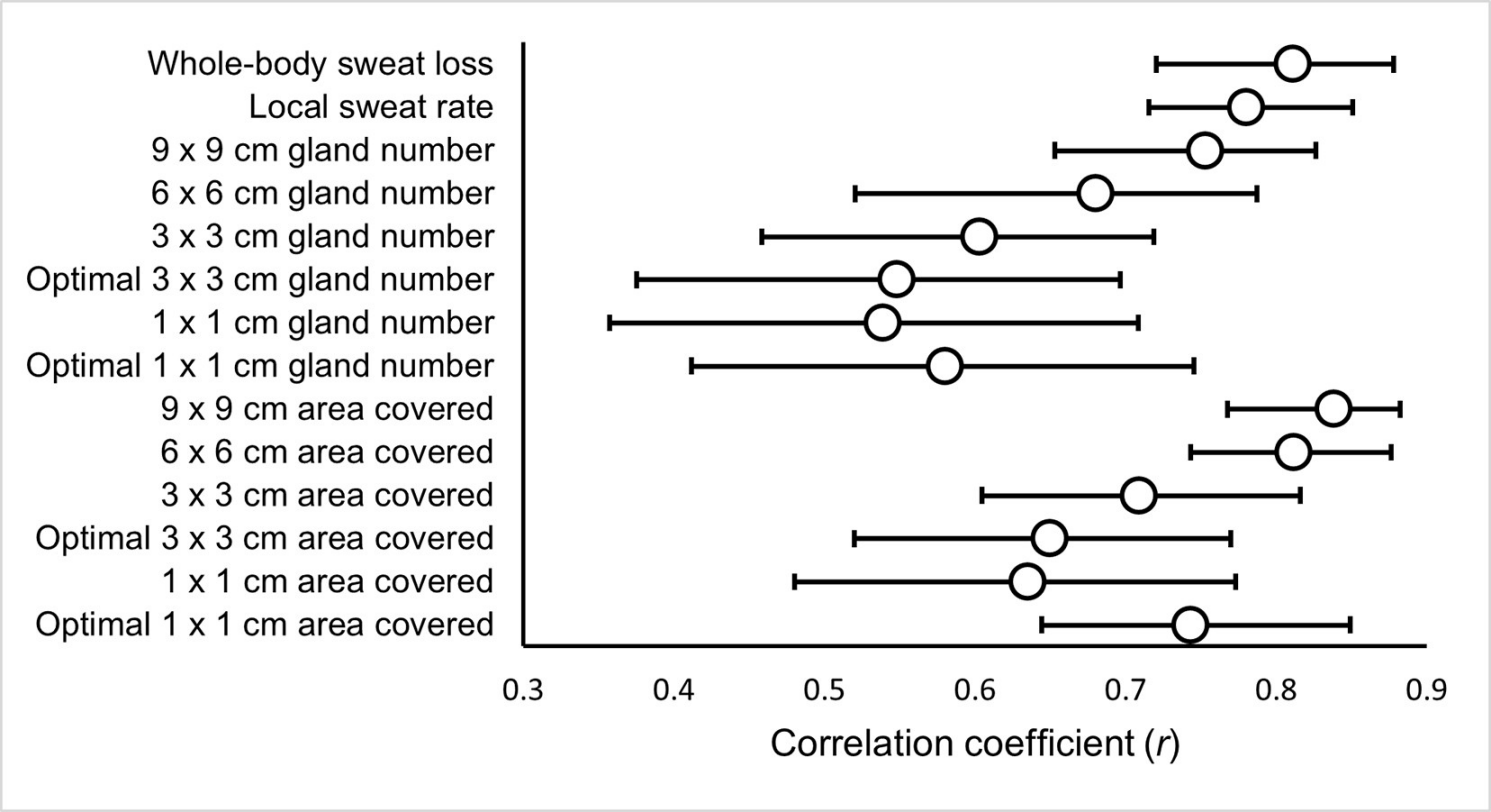
The relationship between relative heat production (Ḣ_prod_) and measures of the sweating response.

**Fig 4:**
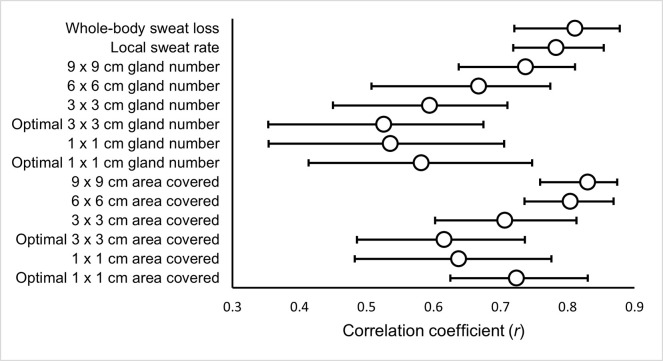
The relationship between absolute evaporative requirement for heat balance (Ė_req_) and measures of the sweating response.

Absolute Ḣ_prod_ (Fig 2), relative Ḣ_prod_ (Fig 3) and absolute Ė_req_ (Fig 4) were significantly correlated with WBSL, LSR and SGA (gland number and surface area covered).

## Discussion

The current study assessed the inter-day reliability of components of thermal balance and multiple measures of the sweating response at rest and various fixed exercise intensities. The pre-to-post exercise body mass change for measurement of WBSL, the absorbent patch technique for the measurement of LSR (Table 2) and the modified iodine-paper technique for the measurement of SGA (both gland counting [Table 3] and area covered [Table 4]), were all found to have greater inter-day reliability during the exercising periods compared to rest. Relative and absolute Ḣ_prod_ and Ė_req_ were found to have very low CVs (Table 1), indicating a controlled environment between the two trials. As expected, all measures of the sweating response had strong or moderate correlations with Ḣ_prod_ (Fig 2) and Ė_req_ (Fig 3; the drivers of sweat production).

It is important to evaluate the systematic bias (unidirectional error) of a test to understand the potential threats to reliability of a measurement technique [35]. For example, in the current study, it was feasible that participants would demonstrate early-phase adaptation to the heat and exercise stimulus across days, or that the user would improve their technique while using manual methods to assess sweating responses. However, there were no systematic biases detected across all but one of the sweating and calorimetry variables, thus indicating that the trials were conducted in a repeatable manner and the techniques used to characterise the participants’ responses were not affected by learning, familiarisation or adaptation effects. Therefore, these techniques, when performed four days apart, can be employed by researchers to control thermal balance and monitor sweating responses among unfamiliarised participants in a laboratory, without concern for systematic adaptations or improvements in the testing procedure. The systematic biases observed for absolute (*P* = .021) and normalised (*P* = .048) sweat gland number using iodine paper at 6 x 6 cm were unanticipated but were largely attributable to two of the participants’ measurements. This is likely to be caused by the natural variation in sweat gland recruitment between testing days, which we discuss in later sections of the current discussion in relation to random error.

Determining the pre vs post difference in body mass is the most basic, yet most common method of measuring sweat loss and, thereby, estimating evaporative cooling [37]. Despite this, we are unaware of a study that has investigated the inter-day reliability of WBSL at rest, and multiple fixed exercise intensities. The CVs for WBSL ranged between 6.0 ± 2.0% and 33.5 ± 23.8%. When all exercise work-rates were combined, this was 7.8 ± 1.8%. The CVs decreased (i.e. reliability improved) with increasing exercise intensity, with resting values demonstrating the poorest reliability (33.5 ± 23.8%) and the highest exercise intensity producing the best reliability (300 W/m^2^ Ḣ_prod_; 6.0 ± 2.0%). These results suggest that WBSL has greater reliability at higher work-rates and, consequently, higher sweat rates. This is consistent with the understanding that a greater afferent stimulus for sweating caused by increasing exercise intensities and ambient temperatures elicits a higher frequency and amplitude of sweat gland recruitment [38] and, thus, more pronounced effect on sweat production [39]. It is likely that the magnitude of stimulus leads to a more sustained effect of sweat production, and therefore more consistent result between trials. On the other hand, the larger CV observed at rest and lower exercise intensities indicates a greater random error or measurement ‘noise’, which can be due to technical (instrument dependent variation), biological variation or human error [25]. The majority of body mass scales have a resolution of ~100 g and therefore smaller body mass changes, as reported at rest, are more difficult to accurately quantify, as they may be just outside the sensitivity of the measurement device. It is important to consider error in relation to ‘signal’ change; for example, WBSL has been reported to vary by 23% due to heat acclimation [22] and by 13% following supplementation of taurine [40]. These signal changes are greater than the measurement noise (CV%) found during exercise in the current study but are smaller than the CV at rest. Therefore, changes in WBSL should be detectable using this technique during exercising periods but might not be identifiable during rest, owing to the inherent noise of the tests between weeks. It should be mentioned that there are alternative techniques for measuring WBSL with greater accuracy, such as a potter balance, however, these are rarely used due to practicality and limited availability [37]. Scales with a resolution of 1 g and accuracy of up to 10 g are also available, though, their high cost can limit their accessibility.

The CVs for LSR, measured using absorbent patches, were 30.1 ± 12.8% overall and 13.8 ± 3.3% for all exercise work rates combined and ranged between 10.1 ± 5.6% and 77.9 ± 39.1%. While the largest CV was also measured during rest (77.9 ± 39.1%), unlike WBSL, these values did not decrease with exercise intensity. The larger CV% found at the highest exercise intensity (Table 1) could be attributed to both technical and human sources of error. For example, at higher sweat rates, the absorbent patches’ adherence to the skin surface can be compromised, which could affect the amount of sweat absorbed by the patch. In addition, it is well-known that sweating varies across regions of the body, with sweat rates typically higher in the upper-back [41]. Even within the same region of the body, sweating can be highly variable, depending on specific locations [15]. Perhaps less-well recognised is the pulsatile manner of sweat gland innervation and secretion, which varies in amplitude, shape and temporal spacing [42]. Therefore, it is possible that the measurements of localised sweat, such as absorbent patch techniques, could be affected by factors such as the timing of measurement and the consistency of patch application on the upper-back. Additionally, the size of the patches applied, and the resolution of the scales used to weigh them, could further affect the results. Overall, these CV% values suggest that the measure of LSR is slightly less reliable than that of WBSL and could make changes in LSR more difficult to detect, particularly during resting conditions across short time periods. However, the CVs are similar to previous findings, where [10] reported inter-day reliability of local sweat rate at the scapula during 90-min of cycling at 75–80% HR_max_ was 14.5%. The ‘noise’ reported during exercise in the current study (13.8%) is markedly less than the expected signal change in LSR on the back with a stimulus such as heat acclimation (~26.7–30%; [23,43,44]).

A poorer reliability at rest was also reflected in the SGA measurements, with resting CV% values ranging from 41.2 ± 22.8% to 70.0 ± 30.5% across the SGA variables and measurement areas. In the exercising periods, however, the CV% values ranged from 15.5 ± 5.4% to 30.0 ± 7.4%, which we attribute to the same reasons provided for other reliability values of resting sweat measures. These values are slightly higher than the intra-trial CV% values (11 ± 10%) previously reported [4], suggesting that the reliability of this technique is slightly less between- than within-day, which could be attributed to greater biological variability across the two separate trial days. Sweat gland number and surface area covered at 9 x 9 cm, optimal 3 x 3 cm and optimal 1 x 1 cm were the most reliable (Tables 2 & 3). For the optimal 3 x 3 cm and 1 x 1 cm areas this was anticipated, because as part of this new approach, the maximum sweat gland density across all regions of the iodine paper was identified. As discussed herein, sweat gland recruitment patterns are pulsatile in nature and vary across anatomical regions [15,41,42]. As such, the pre-designated anatomical regions used in the traditional method are less likely to capture the optimal area of gland activity at any one time-point, leading to increased random error. Searching for the area of optimal gland activity is a more flexible approach, accounting for this variation. The resulting CVs found in this study across exercise intensities supports this. Of course, from a practical perspective, this method is more time-consuming than the traditional measures and researchers should decide whether the incremental improvements (~2%) in reliability warrant the time burden. This decision should be guided by the researcher’s analytical goals [35] but a typical change in SGA as a result of heat acclimation is approximately 27.9% [23] and the CV% values reported here for 9 x 9 cm and optimal 3 x 3 cm and 1 x 1 cm (15.5–17.6%) would permit detection of these changes. In regard to the reliability of the fixed paper sizes of 9 x 9 cm, 6 x 6 cm, 3 x 3 cm and 1 x 1 cm, this was generally poorer with decreasing paper size for both sweat gland number and area of the paper covered in the exercising periods. Moreover, there is variation in the sweat gland number identified per cm^2^ in the larger paper sizes compared to those identified in the smaller ones. This further indicates that SGA is affected by the paper size and, therefore, the results from varying paper sizes are not directly comparable. The explanation for these findings is similar to that provided above, as a larger surface area will more likely include the optimal area of gland activity and account for variability across the body region, which may not be included in smaller paper sizes.

We investigated, for the first time, measurement of the surface area covered, rather than activated sweat gland number. We considered that this could be more reliable than gland number on the basis that co-joining of neighbouring glands could be incorrectly measured as single glands using the counting method, leading to measurement error. We theorised this would most likely be the case at higher sweat rates (i.e. Ḣ_prod_ of 300 W/m^2^), where dots on the paper would be larger and output per gland would be taken into account, as well as number of activated glands. However, findings were equivocal with some paper sizes more or less reliable for gland number vs surface area covered. Nevertheless, these measures performed very similarly in regard to reliability, with the surface area covered marginally less reliable than sweat gland number by approximately ~1.9% across the paper sizes (Tables 2 & 3). Therefore, we conclude that either surface area covered, or the counting method could be used but recognise that the counting method may be preferable for researchers intending to combine sweat gland recruitment with LSRs to determine output per gland [23].

Indeed, it has been shown that progressive recruitment of sweat glands occurs during early exercise in the heat, but at latter stages, LSR is reliant upon sweat production per activated gland [39]. This might also support the current findings, where the 250 and 300 W/m^2^ Ḣ_prod_ exercise intensity produced the most reliable sweat gland measurements. Owing to our study design, the highest work-rates occurred during later stages of exercise and would naturally have recruited most or all of the available sweat glands, leading to greater consistency in the results. Nevertheless, the noise associated with this technique should be considered by potential users and the manual nature of the paper application, particularly during exercise, will inevitably lead to some human error and some unreliability, which might prevent detection of ‘small’ changes in sweat gland activity.

To the best of our knowledge, we are the first to directly report on the inter-day reliability of selected components of thermal balance using partitional calorimetry; namely, absolute and relative Ḣ_prod_ and Ė_req_. Both absolute Ḣ_prod_ (CV% = 1.8 ± 0.5) and Ė_req_ (CV% = 1.7 ± 0.5) and relative Ḣ_prod_ (CV% = 1.9 ± 0.5) and Ė_req_ (CV% = 1.7 ± 0.5) were reliable across all exercise intensities. Given that Ḣ_prod_ and Ė_req_ are determined by a combination of measured variables, such as $\dot{V}O_{2}$ (CV% = 1.7 ± 0.5), Wk and ambient conditions (°C [CV% = 0.2 ± 0.1] and %RH [CV% = 4.3 ± 1.4]), which are commonly used laboratory measures, these results were anticipated. It was necessary to establish the reliability of these variables because of their reported relationship with selected sweat measurements [4,5]. Indeed, as drivers of thermal sweating, consistent control of these thermal balance components is necessary for reliable measurement of sweat responses.

As anticipated, WBSL showed strong significant positive correlations with both absolute Ḣ_prod_ and Ė_req_ (*r*_rm_ = 0.81; *r*_rm_ = 0.81, respectively). There were also moderate to strong, significant positive correlations with Ḣ_prod_ and Ė_req_ for LSR (*r*_rm_ = 0.78; *r*_rm_ = 0.78, respectively). This is consistent with previous reports, where whole-body sweat rate (adjusted *R*^2^ = 0.64; adjusted *R*^2^ = 0.78, respectively; [4,6]) and LSR [5], as measured by the ventilated capsule technique have been associated with absolute Ḣ_prod_ and Ė_req_. Additionally, LSR at the arm (*r* = 0.62; *P* = 0.03; *r* = 0.38; *P* = 0.23) and forehead (*r* = 0.56; *P* = 0.06; *r* = 0.31; *P* = 0.33), determined using absorbent patches, has been moderately correlated with Ė_req_ and Ḣ_prod_ respectively [26]. To expand upon these findings, we evaluated the relationship between other sweat measures, such as SGA, and Ḣ_prod_ and Ė_req_, using an appropriate statistical technique that accounts for repeated observations across the three exercise intensities. Overall, SGA (both gland number and surface area covered), normalised to maximum values, were related to absolute Ḣ_prod_ and Ė_req,_ with surface area covered at 9 x 9 cm (*r*_rm_ = 0.83; *r*_rm_ = 0.83, respectively) and 6 x 6 cm (*r*_rm_ = 0.80; *r*_rm_ = 0.80, respectively) demonstrating the strongest relationships. All other SGA variables demonstrated *moderate* correlations (Figs 2 & 3). The larger iodine paper sizes showed stronger relationships with Ḣ_prod_ and Ė_req_, across both methods of establishing SGA. In addition, the relationships between Ḣ_prod_ and Ė_req_ were consistently greater for surface area covered compared to number of active sweat glands counted. Collectively, these results suggest that larger paper sizes and using the surface area covered method were more consistently associated to the pre-established drivers of thermal sweating. However, all SGA variables related positively with Ḣ_prod_ and Ė_req,_ and, therefore thermal stimuli, and are consequently valid. The reliability of the method should also be considered when deciding upon the method used to establish SGA.

### Limitations, future directions and recommendations

The scales used in this study to measure changes in body mass (for the estimation of WBSL) and the absorbent patches (for estimation of LSR) had resolutions of 50 g and 0.01 g, respectively. Scales with greater resolution may provide slightly different results, especially at rest, as discrete changes in WBSL < 50 g and LSR < 0.01 g would not have been detected in the current study. However, during exercise the results were sufficiently reliable within the limits of the equipment used. The equipment used here can be found in most standard exercise physiology laboratories, which supports the generalisability of the current findings. However, reliability is dependent on the techniques used, which can be affected by various sources of error, caused by factors such as the use of different equipment across laboratories, or the skill and application of different investigators. These factors should be considered when utilising the current data for future purposes.

Both researchers and practitioners, across all applied scientific fields, can use the data reported in the current reliability study to set analytical goals [35] for their research. For example, future users might determine the minimum changes in sweat rate or gland activation as a result of training that are necessary to be deemed beyond the margin of error. Changes in WBSL, LSR and SGA would need to be greater than 7.8%, 13.8% and 15.5–17.6%, respectively to be considered genuine, based on the findings herein. Thus, the current paper can aid the design of future research. Furthermore, researchers in the laboratory or field can use the data reported here to determine the comparability, or perhaps acceptability, of their own techniques. A change greater than the noise of the techniques established in this study should be considered meaningful. Finally, the CV% values reported herein can also be used to determine appropriate sample sizes for future studies. When planning studies, the simple use of a nomogram [45] could be used in conjunction with the current data when an *a-priori* notion of the change in an outcome measure has been established. For example, based on the CV% values reported here, a 10% change in WBSL could be detected with a sample size of approximately 15.

## Conclusion

As was anticipated, all sweating response variables were positively related with Ḣ_prod_ and Ė_req_. Absolute and relative Ḣ_prod_ and Ė_req_ demonstrated inter-day reliability, adequate to control the thermal sweating response. The pre-to-post exercise body mass change for measurement of WBSL, the absorbent patch technique for the measurement of LSR and the various methods of establishing SGA were all also found to have inter-day reliability during the exercising periods, sufficient to detect changes in thermal sweating that might occur following an intervention, such as heat acclimation or dietary manipulation. This was not the case at rest, however, and therefore these methods would be unlikely to be able to detect any changes to the resting sweating response. The modified iodine-paper technique was marginally more reliable at 9 x 9 cm and when the 3 x 3 cm and 1 x 1 cm area of optimal sweat gland density was determined, particularly when measuring the sweat gland number, as opposed to the surface area covered. The larger paper sizes (9 x 9 cm and 6 x 6 cm) had the strongest relationships with Ḣ_prod_ and Ė_req_, especially when SGA was measured using surface area covered. We therefore recommend that to establish SGA at the upper-back, 9 x 9 cm paper sizes are used, with the option of identifying the 3 x 3 cm or 1 x 1 cm optimal areas if deemed necessary. The method of analysis applied (i.e. gland counting or surface area covered) should be chosen based on the research aim.

## Supporting information

S1 File Equations list. (DOCX)

S2 File Analysis of sweat gland activation using ImageJ. (DOCX)

S1 Data (XLSX)
