# Supplementary material for: Measurement of thermal sweating at rest and steady-state exercise in healthy adults: Inter-day reliability and relationships with components of partitional calorimetry
Source: PLoS One. 2022 Dec 1;17(12):e0278652. doi: 10.1371/journal.pone.0278652 (PMC9714830; doi:10.1371/journal.pone.0278652)
Supplement: S1 File — (DOCX) [file pone.0278652.s001.docx]

Supporting information

*Equations list*

$H_{dry skin}= C_{\mathrm{skin}}+R_{\mathrm{skin}}+K_{\mathrm{skin}} [W]$ (equation 7)

H_dry skin,_ dry heat exchange at the skin surface; C_skin_, convection; R_skin_, radiation; K_skin_, conduction

$C_{\mathrm{skin}}+R_{\mathrm{skin}}= \frac{\left( t_{\mathrm{sk}}-t_{0} \right)}{\left( R_{\mathrm{cl}}\frac{1}{h\bullet fcl} \right)}\times A_{D} [W]$ (equation 8)

t_sk_, skin temperature; t_o_, operative temperature; R_cl_, dry heat transfer of clothing; h, combined convective heat transfer coefficient; f_cl_, clothing area factor; A_D_, body surface area

$t_{0}= \frac{h_{r}t_{r}+h_{c}t_{a}}{h_{r}+h_{c}} [W]$ (equation 9)

h_r_, radiative heat transfer coefficient; t_r_, radiant temperature; h_c_, convective heat transfer coefficient; t_a_, ambient air temperature

$h= h_{c}+h_{r} [W/m^{2}/K]$ (equation 10)

$h_{c}=8.3\times v_{\mathrm{air}}^{0.6} [W/m^{2}/K]$ (equation 11)

V_air_, ambient air velocity

$h_{r}= 4 \varepsilon\sigma\frac{A_{r}}{A_{D}}\left( 273.2+\frac{t_{\mathrm{sk}}+t_{r}}{2} \right)^{3} [W/m^{2}/K]$ (equation 12)

ε, non-dimensional emissivity of the body surface; σ, Stefan-Boltzmann constant; A_r_/A_D_, fraction of the body surface participating in radiative heat transfer

$H_{\mathrm{res}}=C_{\mathrm{res}}+E_{\mathrm{res}} [W]$ (equation 13)

H_res_, respiratory heat loss; C_res_, convective respiratory heat loss; E_res_, evaporative respiratory heat loss

$C_{\mathrm{res}}=0.001516\times M\left( 28.56+0.641\times Pa-0.885\times t_{a} \right) [W]$ (equation 14)

M, metabolic energy expenditure; P_a_, vapor pressure of inspired air

$E_{\mathrm{res}}=0.00127\times M(59.34+0.53\times t_{a}-11.63\times Pa [W]$ (equation 15)

$Pa= \frac{6.116441\times{10}^{\left( \frac{7.5911386\times t_{a}}{t_{a}+240.7263} \right)}\times\frac{\%RH}{100}}{10} [kPa]$ (equation 16)

%RH, relative humidity

See Cramer and Jay (2019) for further information regarding partitional calorimetry, these equations and their components.

Cramer MN, Jay O. Partitional calorimetry. Journal of Applied Physiology. 2019;126(2): 267-77.
