# Supplementary material for: Measurement of thermal sweating at rest and steady-state exercise in healthy adults: Inter-day reliability and relationships with components of partitional calorimetry
Source: PLoS One. 2022 Dec 1;17(12):e0278652. doi: 10.1371/journal.pone.0278652 (PMC9714830; doi:10.1371/journal.pone.0278652)
Supplement: S2 File — (DOCX) [file pone.0278652.s002.docx]

Supporting information

*Analysis of sweat gland activation using ImageJ*

- Within the software ImageJ the image was imported (File toolbar – Open – desired image selected).
- The image type was set to 8-bit grayscale (Image toolbar – Type – 8-bit) and converted to binary (Process toolbar – Binary – Make binary). The dots indicating activate sweat glands were now displayed in black on a white background.
- The rectangular box on the left-hand side below the toolbar was selected, which allowed for the desired squared area on the image to be selected for analysis (i.e. 9 cm^2^).
- The number of active sweat glands were counted (Analyze toolbar – Analyze Particles). The lower and upper size limit for the pixel area was set at 10-1000. ‘Display results’, ‘clear results’, ‘exclude on edges’ and ‘record starts’ were selected, alongside ‘Outlines’ under the ‘Show’ menu. Once these setting were established ‘Ok’ was selected and a display box appeared presenting the number of dots counted within the desired area of the image.
- The surface area covered was established (Image toolbar – Adjust – Threshold). A displayed box appeared presenting the percentage of the area covered in dots within the desired area of the image.
- These steps were repeated for analysis of each squared area and each image.
